# Application value of computed tomography and magnetic resonance imaging three-dimensional reconstruction and digital subtraction angiography in percutaneous transhepatic cholangial drainage

**DOI:** 10.3389/fsurg.2022.932901

**Published:** 2023-01-06

**Authors:** Sheng-Li Zhou, Ting-Ju Ji, Xin-Yu Zhou, Lei Xu, Yi Xin, Qiu-Ju Huang

**Affiliations:** ^1^ Department of Imaging, The First People's Hospital of Guannan County, Lian Yun Gang, China; ^2^ Department of Neurology, The First Affiliated Hospital of Kangda College of Nanjing Medical University, Lian Yun Gang City, China; ^3^ Department of Imaging Department, The First People's Hospital of Lian Yun Gang City, Lian Yun Gang City, China

**Keywords:** obstructive jaundice, percutaneous trans-hepatic cholangial drainage, CT and MRI three-dimensional reconstruction, conventional DSA, imaging

## Abstract

**Objective:**

This study aims to explore the application value of computed tomography (CT) three-dimensional (3D) reconstruction, magnetic resonance imaging (MRI) 3D reconstruction, and conventional digital subtraction angiography (DSA) fluoroscopy in percutaneous transhepatic cholangial drainage (PTCD).

**Methods:**

The clinical data of 180 patients with obstructive jaundice requiring PTCD from December 2017 to December 2021 were retrospectively analyzed. Following PTCD, CT 3D reconstruction, MRI 3D reconstruction, and conventional DSA fluoroscopy were conducted, after which the surgical success rates, liver function results, and postsurgical complications were compared.

**Results:**

The puncture accuracies under CT 3D reconstruction, MRI 3D reconstruction, and conventional DSA fluoroscopy were 90.0% (54/60), 96.7% (58/60), and 80% (48/60), respectively. The degree of jaundice and epigastric discomfort was relieved in all three groups after surgery, while a significant reduction in the levels of total bilirubin and direct bilirubin was observed relative to the levels before surgery (*P* < 0.05). The incidences of complications in the CT 3D reconstruction, MRI 3D reconstruction, and conventional DSA fluoroscopy groups were 6.7% (4/60), 3.3% (2/60), and 13.3% (8/60), respectively, and the differences among the three groups were statistically significant (*P* < 0.05).

**Conclusion:**

Conducting conventional enhanced CT and MRI scans in patients before surgery might be more practical than the conventional puncture method. Among the methods under study, MRI 3D reconstruction was found to be safer and more feasible than CT 3D reconstruction and conventional DSA fluoroscopy in PTCD. MRI 3D reconstruction could reduce the degree of jaundice, improve the success rate of surgery, reduce the incidence of complications due to surgery, and improve the patients’ tolerance to surgery.

## 1. Introduction

Malignant obstructive jaundice (MOJ) is a type of jaundice caused by a biliary obstruction resulting from malignant tumors such as pancreatic head cancer and cholangiocarcinoma. Due to poor biliary drainage, the persistent malignant biliary obstruction may lead to jaundice, coagulation dysfunction, and/or liver and kidney insufficiency. Palliative surgery is therefore the only option for patients with MOJ. Percutaneous transhepatic cholangial drainage (PTCD) combined with biliary stent implantation is a simple and effective treatment that can relieve jaundice and obstruction, providing an opportunity to restore biogenic biliary drainage after surgery. This procedure also causes minimal damage to tissues and organs, which results in rapid postoperative recovery. At present, PTCD has been widely adopted and promoted in China and abroad and has become the main method for the treatment of MOJ (1–3). In recent years, PTCD guided by imaging equipment [x-ray, ultrasound, and computed tomography (CT)] has gradually become the preferred method for resolving malignant biliary obstructions. However, due to the limitations of surgical vision, the surgeon may need to puncture repeatedly, which may lead to serious complications such as bleeding and pneumothorax. Therefore, in the present study, the applications of CT and magnetic resonance imaging (MRI) three-dimensional (3D) positioning combined with digital subtraction angiography (DSA) fluoroscopy for puncturing in PTCD were investigated to further improve the puncture success rate and reduce the incidence of complications (4–9).

## 2. Materials and methods

### 2.1. General characteristics

#### 2.1.1. Clinical data

The clinical data of 180 patients with obstructive jaundice requiring PTCD from December 2017 to December 2021 were retrospectively analyzed. The patients were divided into three groups: CT 3D reconstruction, MRI 3D reconstruction, and conventional DSA fluoroscopy, with 60 cases in each group. There were 108 cases with hepatobiliary malignant tumors and 72 cases with malignant pancreatic tumors. Among them, 100 patients were men and 80 were women. Patients’ age ranged from 40 to 75 years, with an average age of 61.05 ± 5.28 years. The differences in age, sex, type of cholangiocarcinoma (Bismuth–Corlette), pancreatic tumor location, tumor size, and degree of the extrahepatic bile duct and intrahepatic bile duct dilatation were not statistically significant among the three groups (*P* < 0.05). Preoperative informed consent was signed by the patients or their immediate family.

#### 2.1.2. Entry criteria for study subjects

The eligibility criteria are as follows: (1) a history of digestive malignancies, obstructive jaundice diagnosed by imaging examination (ultrasound, CT, or MRI), with clear indications for PTCD and (or) biliary stent implantation; (2) PTCD and (or) biliary stent implantation; and (3) all punctures by a right-sided approach.

#### 2.1.3. Group criteria

The patients were divided into two groups by the following criteria: (1) Observation group: CT and MR 3D reconstruction of the biliary system was performed before the operation and biliary puncture was performed in DSA after calculating the reconstructed image; and (2) Control group: CT and MR 3D reconstruction was not performed before surgery and biliary puncture was performed in DSA according to the classical method.

### 2.2. Operation methods

#### 2.2.1. Observation groups

Preoperative routine examinations of electrocardiograms, electrolytes, and liver and kidney functions were performed to rule out contradications to PTCD. Enhanced CT scanning and magnetic resonance cholangiopancreatography (MRCP) of the upper abdomen were conducted for the observation group. The CT and MRI 3D reconstruction techniques were adopted for postprocessing to determine the site of obstruction and depth of the puncture and to measure the width of the bile duct and select an appropriate puncture site. Each method is described in more detail in the following.

##### 2.2.1.1. CT 3D reconstruction examination method

A 64-row, 128-slice CT scanner was used to determine the dilatation of the intra- and extrahepatic bile ducts and the puncture site. A thin-section scan was conducted on the puncture site, after which 3D reconstruction was adopted to determine the specific site, direction, and depth of the puncture. After local anesthesia was administered, the puncture was performed using a puncture needle with a predetermined direction and depth. After reaching the position, the needle core was withdrawn, the drainage tube was flushed with gentamicin saline solution daily, and a drainage tube was finally indwelled. The drainage fluid could be utilized for bacterial culture if desired. It is recommended that anti-infection measures, hemostasis support, anti-inflammation measures, liver protection measures, nutritional support, and treatment of symptoms should be implemented after the operation.

##### 2.2.1.2. MRI 3D reconstruction examination method

A Siemens 3.0 T superconducting magnetic resonance scanner was adopted. Each patient fasted for 6–8 h before the examination. The conventional MRI sequence was the TSE sequence with the conduction of plain scanning and enhanced scanning (in a few cases, no enhanced scan was conducted). A single-excitation. T2-weighted sequence with breath-holding, respiratory gating, and fat suppression techniques was adopted in the MRCP with a rotation at 15° intervals in the lateral position. A total of 10–12 blocks were scanned and imaged on a workstation for 3D MIP reconstruction. The images of the MRI plain scan and MRI enhanced scan sequences were observed together with the MRCP raw images and MIP 3D reconstructed images.

##### 2.2.1.3. Conventional DSA fluoroscopy method

The PTCD procedure was conducted under the guidance of an x-ray. Local puncture anesthesia was used, and the patient was placed in a supine or left-lying position with the right upper limb placed behind the head. Under DSA, the eighth intercostal space in the right axillary midline was adopted as the puncture point. The puncture needle was pointed at the inferior border of the 11th thoracic vertebra, and the tail of the needle was kept horizontal with the needle at approximately 2 cm near the spine. Under the guidance of C-arm x-ray fluoroscopy, the puncture needle was inserted into the dilated target bile duct.

**Figure 1:**
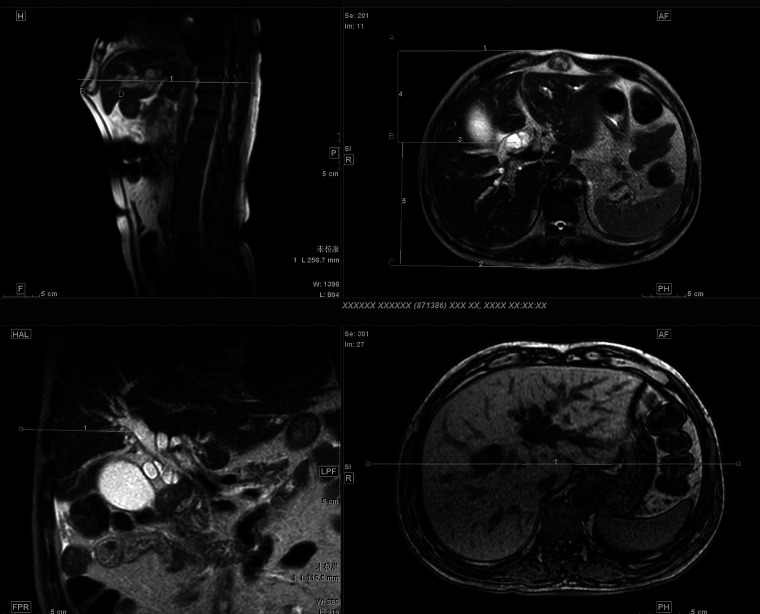
Four points ABCD were selected, where AB represents the distance between the puncture point on the body surface and the anterior chest wall, point B represents the puncture point on the body surface, BC is the distance from the puncture point on the body surface to the posterior chest wall, and point D is the arrival point of the puncture needle for bile duct dilatation. The puncture level BD was determined, and the distances of AB, BC, and BD were measured.

For the selection of the puncture site for the PTCD approach, clinically, the axillary midline and subxiphoid approaches were mostly adopted, with the needle tip adjusted under fluoroscopy to point to the 11th thoracic vertebrae, keeping the tail of the needle horizontal and the needle close to the spine with a distance of approximately 2 cm. This approach has a low puncture success rate and often requires multiple punctures. However, in the present study, the patient underwent preoperative conventional enhanced CT and MRI scans, and the level, site, and depth of the puncture, along with the angle of needle entry, were determined based on the 3D reconstructed images. Finally, based on the above data, the success rate of the puncture under DSA was high, with observed avoidance of vessels and a reduction in the incidence of bleeding. Four points (A, B, C, D) were selected; here, AB represents the distance between the puncture point on the body surface and the anterior chest wall, point B represents the puncture point on the body surface, BC denotes the distance from the puncture point on the body surface to the posterior chest wall, and point D indicates the arrival point of the puncture needle for bile duct dilatation. After marking, the puncture depth BD was determined and aligned with the level of the punctured vertebra. The 10th thoracic vertebra level is shown in Figure 1. The puncture level BD was determined, and the distances AB, BC, and BD were measured (see Figure 1): the distance AB was 11.9 cm, the distance BC was 12.5 cm, and the puncture depth BD was 10.2 cm. Through precise measurement of the puncture site, the puncture depth and the angle of entry at the puncture level, the direction of needle entry, and the distance of needle entry could be marked on the 3D reconstruction image.

### 2.3. Observation indicators

The PTCD procedure was conducted with the help of CT 3D reconstruction, MRI 3D reconstruction, and conventional DSA fluoroscopy. The surgical success rate, liver function, and incidence of complications among the three groups were compared. The main postoperative complications were postoperative biliary tract infection, recurrent biliary tract infection, and restenosis.

### 2.4. Statistical methods

The SPSS 25.0 software package was adopted for statistical processing of the data. The Shapiro–Wilk test was used for the evaluation of the normal distribution of the measurement data, while the M(Q1, Q3) was used to describe the data that did not satisfy the normal distribution. The Wilcoxon signed rank-sum test was adopted to compare the levels of total bilirubin and direct bilirubin before and after the operation. The countable data were expressed as a percentage (%) and tested by a *χ*^2^ test. *P* < 0.05 was considered statistically significant.

## 3. Results

### 3.1. Comparison of the success rate in the three groups after percutaneous transhepatic cholangial drainage

The puncture accuracy achieved using the MRI 3D reconstruction technique was 96.7% (58/60), while that using the CT 3D reconstruction technique was 90.0% (54/60); both of these were higher than the puncture accuracy of 80% (48/60) using conventional DSA.

### 3.2. Comparison of liver function recovery among the three groups

Before the operation, the serum levels of total bilirubin and direct bilirubin in the three groups increased significantly; on the seventh day after the operation, the levels of total bilirubin and direct bilirubin decreased significantly in all three groups. The differences in levels among the three groups before and after the operation are listed in Table 1. The reduction in the level of total bilirubin in the conventional PTCD group was lower than that in the other two groups, while the difference in the reduction in the level of direct bilirubin was not statistically significant among the three groups, as shown in Table 2.

**Table 1:** Liver functions in 180 patients before and after the operation [M(Q1, Q3), **μ**mol/L].

Item	Before the operation	After the operation	*Z*	*P*
Total bilirubin	224.75 (110.95–326.5)	110.50 (64.28–201.45)	8.980	0.000
Direct bilirubin	227.80 (147.20–348.3)	99.65 (51.10–189.16)	9.828	0.000

**Table 2:** Comparison of the reduction in the levels of bilirubin among the three groups [M(Q1, Q3), **μ**mol/L].

Item	*n*	Reduction in total bilirubin	Reduction in direct bilirubin
CT three-dimensional reconstruction	60	110.30 (36.48–165.06)	108.10 (30.10–164.16)
Conventional DSA group	60	29.10 (−20.60,86.10)	105.80 (48.00,186.80)
MRI three-dimensional reconstruction	60	143.40 (66.20,212.90)	139.20 (55.00,212.100)
*H*	–	33.071	2.348
*P*	–	0.000	0.309

The comparison of reduction in total bilirubin between the CT three-dimensional reconstruction group and the conventional DSA group (*Z* = −4.064, *P* < 0.001). The comparison of reduction in total bilirubin between the MRI three-dimensional reconstruction group and the conventional DSA group (*Z* = −5.523, *P* < 0.001).

### 3.3. Comparison of the incidence of postoperative complications among the three groups

The incidences of complications in the CT 3D reconstruction group, the MRI 3D reconstruction group, and the conventional DSA fluoroscopy group were 6.7% (4/60), 3.3% (2/60), and 13.3% (8/60), respectively; moreover, the differences among the three groups were statistically significant (*P* < 0.05). The incidence of bile duct infection was significantly higher in the conventional DSA fluoroscopy group than those in the other two groups (*P* < 0.05), while the difference in the incidence of bile duct infection between the CT 3D reconstruction group and the MRI 3D reconstruction group was not statistically significant (*P* > 0.05). The differences in the recurrence rate of biliary restenosis and bile duct infection among the three groups were also not statistically significant (*P* > 0.05).

## 4. Discussion

As improvements to medical treatments progress, PTCD technology continues to develop. The PTCD procedure is less invasive and facilitates quick patient recovery after surgery. Compared with other methods, PTCD has significant advantages and is thus widely adopted by physicians and highly praised by patients, achieving good results in treating obstructive jaundice. Generally, PTCD is conducted under the guidance of ultrasonography or CT in combination with x-ray fluoroscopy (10–15). Under the guidance of DSA fluoroscopy, the details of the patient's intra- and extrahepatic bile ducts can be directly observed to facilitate the smooth passage of the guidewire. It also assists in the selection of the optimal drainage tube implantation position. In addition, it reduces the difficulty of internal and external bile duct drainage (and of stent implantation for bile duct drainage) to a certain extent, which has many advantages. However, puncture under DSA fluoroscopy is somewhat blinded, and the success rate of the first puncture is low. Patients also need to be exposed to radiation for long periods and multiple punctures are often required, possibly resulting in additional complications and high risks. Although ultrasound-guided puncture localization can improve the success rate of the first puncture to some extent, due to the limitations of the ultrasound image field and the low image resolution, it is impossible to observe the guidewire and catheter in real time following a successful puncture and difficult to guide the placement of the biliary stent; this causes a range of problems during the placement of the guidewire and catheter, making it a time-consuming and laborious process. A series of problems may also occur in the patient during the procedure.

In the present study, using a combination of CT 3D positioning, MRI 3D positioning, and DSA fluoroscopy, as well as PTCD, conventional scanning and enhanced scanning were conducted on the patients before the operation. The site, level, depth, and angle of the puncture were determined through 3D image reconstruction, after which the puncture was made on the DSA according to the determined values. This not only improved the success rate of the puncture but also significantly reduced the incidence of bleeding by avoiding damage to the vessel through real-time observation.

In addition, MRI 3D reconstruction was found to be safer and more feasible than CT 3D reconstruction and conventional DSA fluoroscopy. Compared with simple x-ray fluoroscopic guidance, the MRI 3D reconstruction guidance mode not only compensated for the blindness of the puncture but also significantly reduced the incidence of complications. This further improved the puncture success rate, effectively shortened the procedure duration, greatly reduced the required x-ray radiation dose, significantly reduced the risk associated with the procedure, and significantly alleviated pain in the patient. Compared with CT 3D reconstruction and conventional DSA fluoroscopy, MRI 3D reconstruction had a unique role in PTCD. In addition, MRI 3D reconstruction might help in selecting the appropriate puncture site and route, visually exploring the overall image of the biliary tree, improving the success rate of one-time punctures, and ensuring unobstructed bile drainage.

In conclusion, the MRI 3D reconstruction technique in PTCD was not only safe but also effectively shortened the therapeutic duration and reduced the number of punctures required, accordingly yielding a higher success rate. It can additionally reduce the incidence of surgical complications and alleviate pain in the patient, increasing their likelihood of being able to tolerate the pain and discomfort during the operation.

## Data Availability

The original contributions presented in the study are included in the article/Supplementary Material, further inquiries can be directed to the corresponding author.

## References

[1] Qi S Yan H. Effect of percutaneous transhepatic cholangial drainag+radiofrequency ablation combined with biliary stent implantation on the liver function of patients with cholangiocarcinoma complicated with malignant obstructive jaundice. Am J Transl Res. (2021) 13(3):1817–24. PMID: 33841706. 33841706 PMC8014363

[2] Bao G Liu H Ma Y Li N Lv F Dong X The clinical efficacy and safety of different biliary drainages in malignant obstructive jaundice treatment. Am J Transl Res. (2021) 13(6):7400–5. PMID: 34306512. 34306512 PMC8290822

[3] Sarkodie BD Botwe BO Brakohiapa E. Percutaneous transhepatic biliary stent placement in the palliative management of malignant obstructive jaundice: initial experience in a tertiary center in Ghana. Pan Afr Med J. (2020) 37:96. 10.11604/pamj.2020.37.96.20050 33425129 PMC7757328

[4] Sha J Dong Y Niu H. A prospective study of risk factors for in-hospital mortality in patients with malignant obstructive jaundice undergoing percutaneous biliary drainage. Medicine. (2019) 98(15):e15131. 10.1097/MD.0000000000015131 30985679 PMC6485810

[5] Duan F Cui L Bai Y Li X Yan J Liu X. Comparison of efficacy and complications of endoscopic and percutaneous biliary drainage in malignant obstructive jaundice: a systematic review and meta-analysis. Cancer Imaging. (2017) 17(1):27. 10.1186/s40644-017-0129-1 29037223 PMC5644169

[6] Huang P Zhang H Zhang X-F Lv W Lou S. Comparison of endoscopic ultrasonography guided biliary drainage and percutaneous transhepatic biliary drainage in the management of malignant obstructive jaundice after failed ERCP. Surg Laparosc Endosc Percutan Tech. (2017) 27(6):e127–31. 10.1097/SLE.0000000000000485 29206804

[7] Wang Y Lyu Y Li T Wang B Cheng Y. Comparing outcomes following endoscopic ultrasound-guided biliary drainage versus percutaneous transhepatic biliary drainage for malignant biliary obstruction: a systematic review and meta-analysis. J Laparoendosc Adv Surg Tech. (2022) 32(7):747–55. 10.1089/lap.2021.0587. 34677099

[8] Filipović AN Mašulović D Zakošek M Filipović T Galun D. Total fluoroscopy time reduction during ultrasound- and fluoroscopy-guided percutaneous transhepatic biliary drainage procedure: importance of adjusting the puncture angle. Med Sci Monit. (2021) 27:e933889. 10.12659/MSM.933889 34802031 PMC8614062

[9] Staron R Rzucidło M Macierzanka A Krawczyk M Gutkowski K Krupa L. Unresectable malignant obstructive jaundice: a 2-year experience of EUS-guided biliary drainage. BMJ Support Palliat Care. (2021). 10.1136/bmjspcare-2020-002335 33653733

[10] Kongkam P Orprayoon T Boonmee C Sodarat P Seabmuangsai O Wachiramatharuch C ERCP plus endoscopic ultrasound-guided biliary drainage versus percutaneous transhepatic biliary drainage for malignant hilar biliary obstruction: a multicenter observational open-label study. Endoscopy. (2020) 53(01):55–62. 10.1055/a-1195-8197 32515005

[11] Comparison of endoscopic ultrasonography guided biliary drainage and percutaneous transhepatic biliary drainage in the management of malignant obstructive jaundice after failed ERCP: erratum. Surg Laparosc Endosc Percutan Tech. (2018) 28(2):133. 10.1097/SLE.0000000000000515 29613967

[12] Nennstiel S Treiber M Faber A Haller B von Delius S Schmid RM Comparison of ultrasound and fluoroscopically guided percutaneous transhepatic biliary drainage. Dig Dis. (2018) 37(1):77–86. 10.1159/000493120 30253406

[13] Guohua L Changan D Feng L Xinchang D Bin L. Clinical value of ultrasound-guided percutaneous transhepatic biliary drainage in the treatment of obstructive jaundice. Chin Med J. (2016) 51(05):103–4.

[14] Xiong B Analysis of the choice of approach for percutaneous transhepatic biliary drainage under ultrasound guidance in 225 cases. Chinese J Interv Radiol. (2013) 1(01):53–7.

[15] Na Y Bo W Wei H Gang W. The clinical value of ultrasound - guided percutaneous transhepatic biliary drainage in the treatment of obstructive jaundice. Chinese J Interv Imaging Ther. (2012) 9(07):494–6.

